# Supplementary material for: PKCα Activation via the Thyroid Hormone Membrane Receptor Is Key to Thyroid Cancer Growth
Source: Int J Mol Sci. 2024 Nov 13;25(22):12158. doi: 10.3390/ijms252212158 (PMC11594262; doi:10.3390/ijms252212158)
Supplement: Supplementary file 1 [file ijms-25-12158-s001.zip › ijms-3205675-supplementary.pdf]

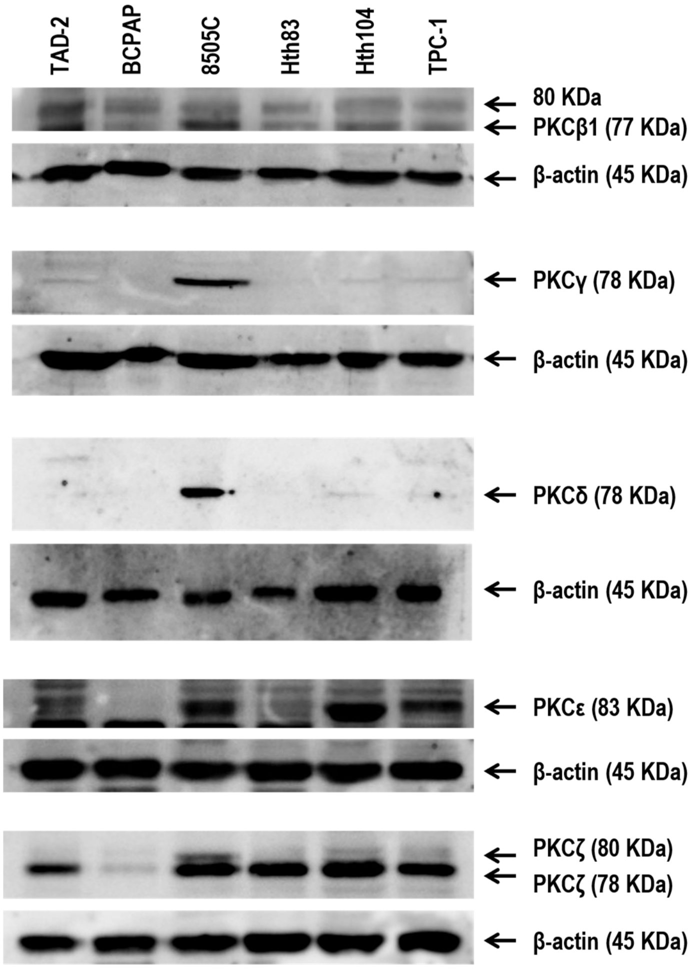

Supplementary Figure S1: PKC expression in TC cells. PKCβ, PKCγ PKCδ, PKCε and PKCζ protein levels in in TAD-2 and in epithelial TC cells as determined by Western blot. β-actin was used as loading control.

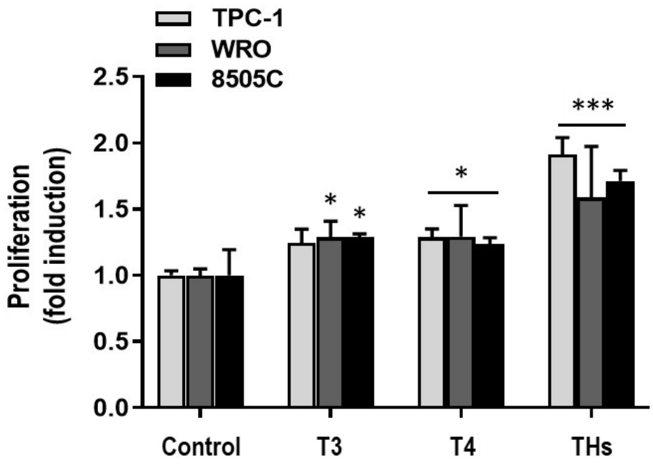

Supplementary Figure S2: Individual or combined action of THs on TC cell proliferation. TPC-1, WRO and 8505C TC cell lines untreated (Control) or incubated with 1 nM T3 alone, 100 nM T4 alone (physiological concentrations), or T3 and T4 in combination (THs) for 48 h. Cell Titer Blue assay determined the number of live cells at each dose. Data are shown as mean ± SD. ***$p<0.0001$, *$p<0.05$ respect to control.

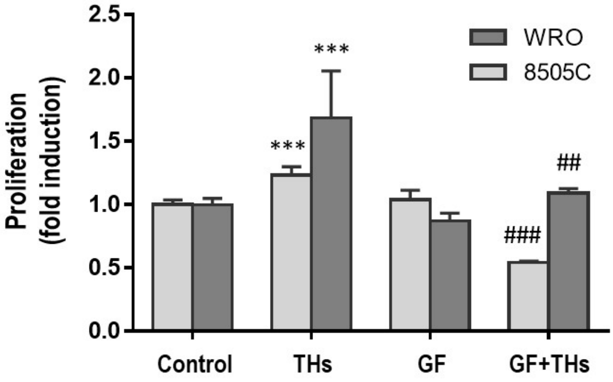

Supplementary Figure S3: PKC inhibitors in hormone-induced proliferation in TC cells. WRO and 8505C cells were pretreated or not (Control) with 5 µM GF109203X and then incubated with 1 nM T3 and 100 nM T4 (physiological concentrations) in combination for 48 h. Cell Titer Blue assay determined the number of live cells at each dose. Data are shown as mean ± SD. ***p<0.0001 respect to control; ###p<0.0001 and ##p<0.005 respect to T3/T4 treated cells.

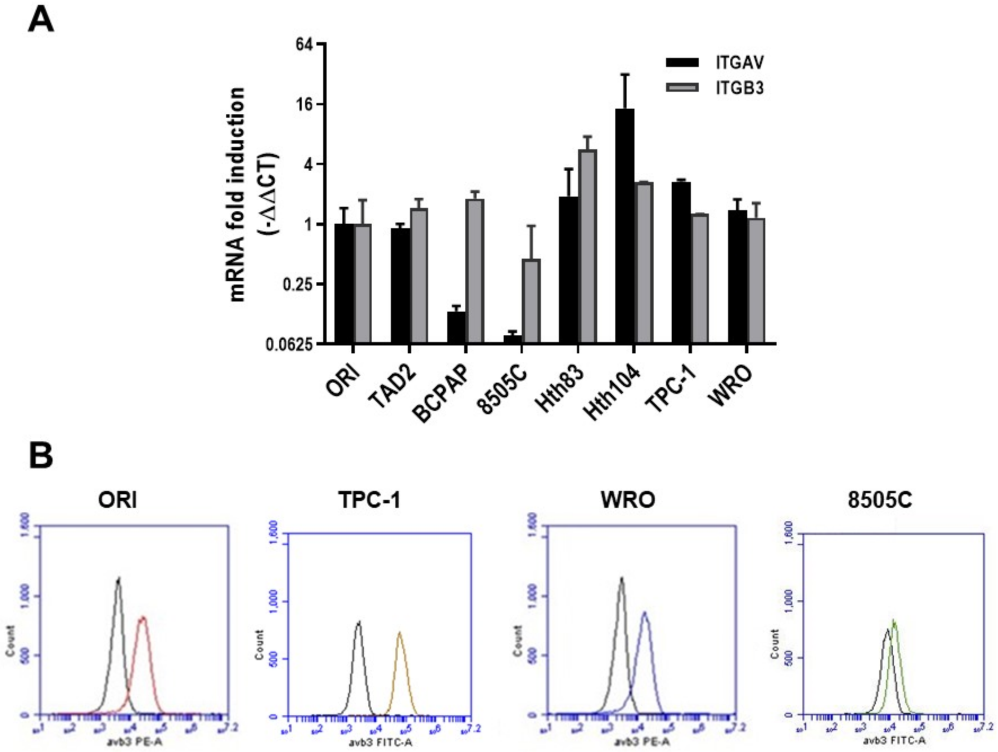

Supplementary Figure S4: Integrin αvβ3 expression in TC cells. A. mRNA levels of integrin αv and β3 were analyzed by qPCR in thyroid cells. Gene expression was normalized to β2-microglobulin gene using the ΔΔCt method. Data are expressed as mean S.E. of three independent experiments. **B.** αvβ3 integrin levels in ORI, TPC-1, WRO and 8505C TC cells was also evaluated by flow cytometry analysis.

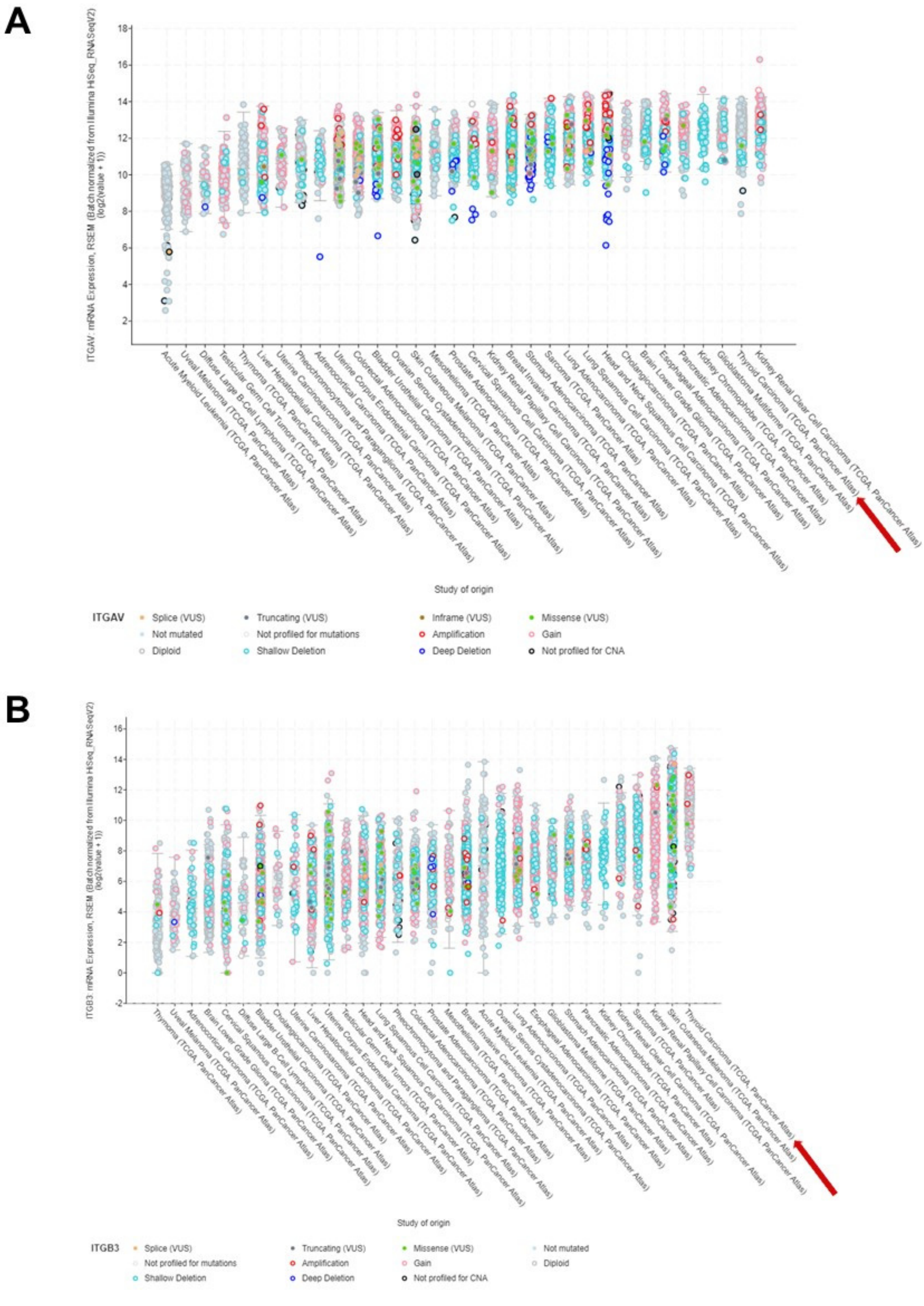

Supplementary Figure S5: Analysis of integrin αv and β3 mRNA expression in cancer patients. The RNA Seq mRNA expression levels of ITGAV **(A)** and ITGB3 **(B)** in multiple in human thyroid tumors with different types of cancer were obtained from TGCA database using cBioPortal.

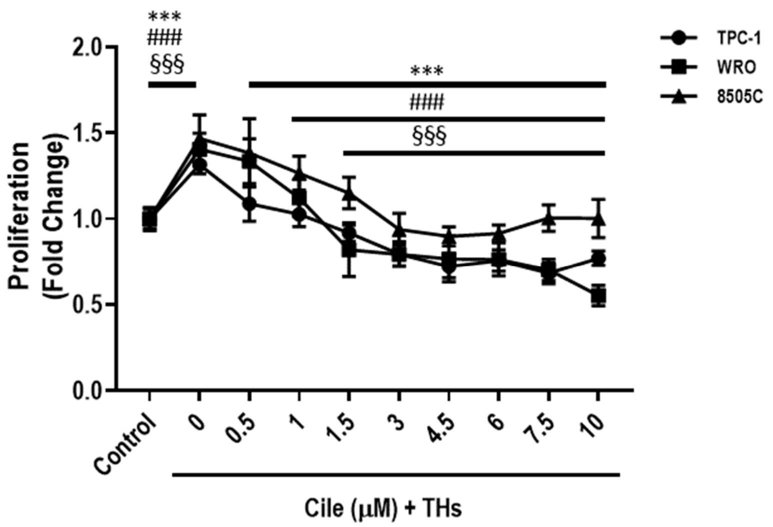

Supplementary Figure S6: Dose response inhibition of THs effect on TC cells proliferation by Cilengitide. TPC-1, WRO and 8505C cells were grown in the absence of FBS and then pretreated or not (Control) with increasing concentrations of Cile and then incubated with 1 nM T3 and 100 nM T4 in combination (physiological concentrations, THs) for 48 h. By Cell Titer Blue assay, the number of living cells was determined with each treatment. The commercial kit (Promega) was used as indicated by the manufacturer. The mean ± SD is shown. ***$p<0.0001$ respect to THs (TPC-1), ###$p<0.0001$ respect to THs (WRO), §§§$p<0.0001$ respect to THs (8505C).

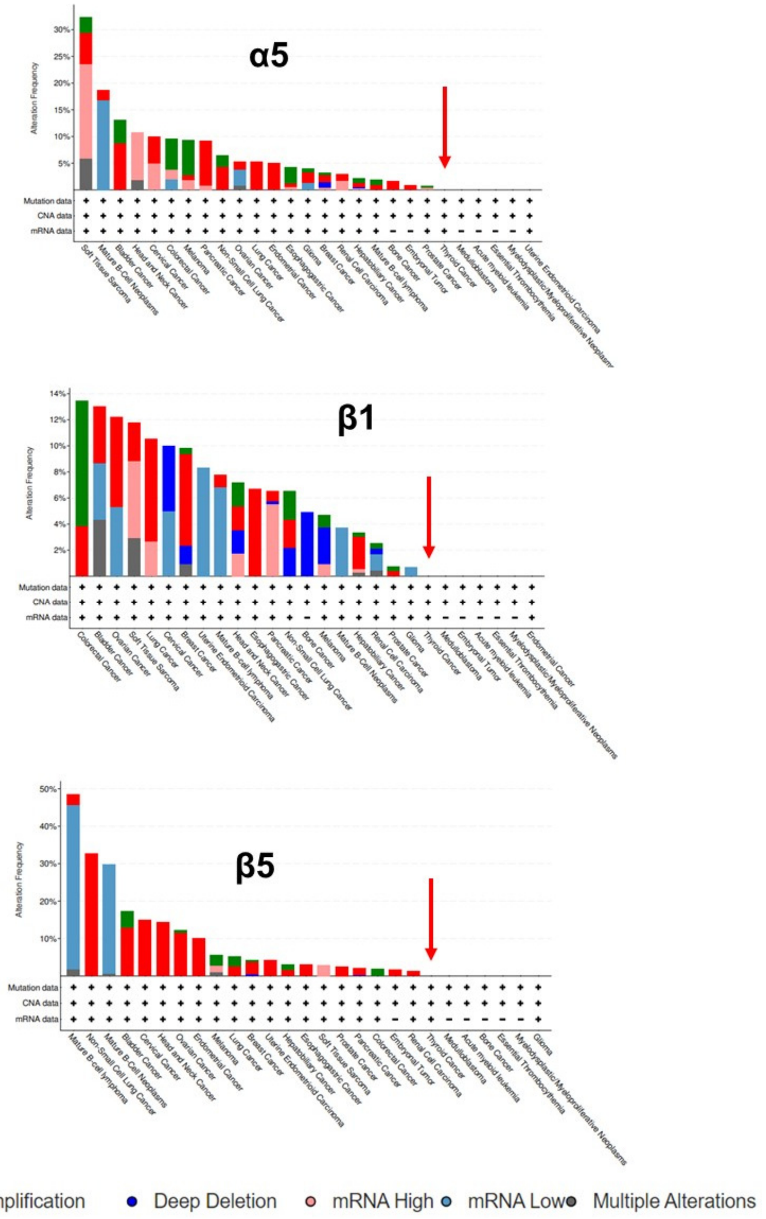

Supplementary Figure S7: Analysis of mRNA expression of Cilengitide target integrins in cancer patients. The RNA Seq mRNA expression levels of ITGA5 **(A)**, ITGB1 **(B)** and ITGB5 **(C)** in multiple cohorts of patients with different types of cancer were obtained from TGCA database using cBioPortal.
